# Action Observation Combined With Motor Imagery Training to Improve Motor Function in People With Stroke: Systematic Review and Meta-Analysis

**DOI:** 10.2196/75705

**Published:** 2025-10-27

**Authors:** Pei Sun, Xiao Liang, Xin Zhang, Mei Huang, Xiao Zhang, Chunping Ni

**Affiliations:** 1 Department of Basic Nursing, School of Nursing, Air Force Medical University, 169 West Changle Rd, Xi'an, 710032, China, 86 02984711721; 2 School of Nursing, Shaanxi University of Chinese Medicine, XianYang, China

**Keywords:** action observation, motor imagery, limb motor function, systematic review, meta-analysis, stroke

## Abstract

**Background:**

Action observation combined with motor imagery (AO+MI) training is considered a potentially effective approach for improving motor function in patients after stroke. Therefore, it is important to review and analyze the existing research evidence of its effectiveness.

**Objective:**

This study aims to evaluate the effectiveness of AO+MI training on the limb motor function of patients with stroke.

**Methods:**

A systematic search was conducted in PubMed, Cochrane Library, Web of Science, Embase, Proquest, Physiotherapy Evidence Database, ClinicalTrials.gov, and ChiCTR. The last search was performed in June 2025. Three reviewers independently screened the articles, and 2 reviewers extracted data. Quality assessments of randomized controlled trials were done using the Cochrane Risk-of-Bias Tool. The certainty of evidence was evaluated with GRADEpro GDT (Evidence Prime, Inc). A meta-analysis was performed using RevMan 5.3 (The Cochrane Collaboration) software and Stata software.

**Results:**

A total of 13 articles were included with 399 patients with stroke. The results of the meta-analysis showed that compared with routine rehabilitation, AO+MI could improve the upper extremity function (standard mean difference [SMD]=1.02, 95% CI 0.28‐1.75; *P*=.007) and the lower extremity function (SMD=6.31, 95% CI 4.75‐7.87; *P*<.001) of patients with stroke. There was no significant difference between AO+MI and routine rehabilitation for improving activities of daily living (SMD=0.06, 95% Cl –0.35 to 0.47; *P*=.06). AO+MI could promote the recovery of upper extremity function in patients compared with MI independently (SMD=0.97, 95% Cl 0.13‐1.80; *P*=.02). There was no significant difference between synchronous combination and asynchronous combination in upper extremity function rehabilitation of patients after stroke (SMD=−1.04, 95% Cl –2.56 to 0.48).

**Conclusions:**

AO+MI can improve the motor function of limbs and can be considered an effective limb rehabilitation therapy for patients after a stroke.

## Introduction

Stroke represents a major cause of long-term adult disability worldwide [1]. About 60% of patients with stroke will have different degrees of limb dysfunction [2], which will not only have a significant impact on the quality of life of patients and caregivers but also increase the additional economic burden [3,4].

Action observation (AO) refers to watching human movement either via a prerecorded video or a live demonstration [5]. Motor imagery (MI) is defined as the mental simulation of a given movement that is internally reproduced in the brain without any actual motor output [6]. Both AO and MI can activate the neurons in the motor area [7-9], improve brain structure, and positively affect the motor behavior and performance of the patients [10-12], which are considered effective interventions to promote motor learning and rehabilitation [13,14]. In recent years, systematic reviews have been conducted to analyze the effectiveness of AO or MI on limb function rehabilitation of patients with stroke. Silva et al [6] synthesized data from 6 randomized controlled trials and found that, compared to other therapies, MI is more beneficial for improving poststroke gait (walking speed) at the end of treatment (standardized mean difference [SMD]=0.44). Barclay et al [15] systematic review revealed moderate-quality evidence indicating that MI combined with other treatments improves upper extremity activity (SMD=0.66) and reduces upper extremity impairment (SMD=0.59) in adult survivors of stroke with deficits in upper extremity activity. Borges et al [16] meta-analysis demonstrated that AO improved arm function (SMD=0.39) and enhanced hand function (mean difference=2.76). Peng et al [17] meta-analysis results indicated that, compared to control treatments, AO had a moderate-to-large effect size on walking outcomes (Hedges *g*=0.779), a large effect size on gait velocity (Hedges *g*=0.990), and a moderate-to-large effect size on activities of daily function (Hedges *g*=0.728). When patients with hemiplegia after stroke cannot complete rehabilitation training, AO or MI can replace or supplement traditional rehabilitation training and become a feasible therapy.

The combined intervention of AO and MI (AO+MI) is typically defined as observing an action while simultaneously imagining the feelings associated with performing it [18]. More recently, it has been found that the brain regions involved in MI and AO overlap extensively with each other and with the regions involved in motor execution, so combined use may promote cortical activation in the premotor, rostral parietal, and somatosensory areas [19,20]. A population-independent meta-analysis revealed that AO+MI increased corticospinal excitability compared to AO and control interventions [21]. The training measures of AO+MI have been applied to healthy participants [22], Parkinson disease [23], and limb injuries [24]. A systematic review study on populations with Parkinson disease found that AO+MI can improve individuals’ freezing of gait, speed, physical function, and balance [25]. Some studies [26,27] have applied AO+MI to rehabilitating limb function in patients with stroke. However, most of these studies are small sample trials, and there is heterogeneity among the results of the studies, so the validity is unclear.

The combination of AO and MI has 2 modes: synchronous and asynchronous. In synchronous mode, participants observe human movement, and at the same time, imagine themselves executing either the same or a different action. In asynchronous mode, AO happens at a different time than MI [28]. Some scholars believe synchronous AO+MI has unique advantages [5], but others argue that it is unclear whether synchronous indeed improves performance beyond asynchronous [29-31].

Therefore, our systematic review and meta-analysis aim to explore (1) whether AO+MI has more advantages in limb function rehabilitation of patients with stroke compared with routine rehabilitation, (2) whether AO+MI has an advantage in limb function rehabilitation of patients with stroke compared with their independent use, and (3) whether there are differences in limb function rehabilitation of patients with stroke between synchronous AO+MI and asynchronous AO+MI. Through this review, we discuss the feasibility and effectiveness of applying AO+MI in the rehabilitation of limb function in patients with stroke to provide evidence-based evidence for clinical practice. We hope that this systematic review can provide clinical rehabilitation therapists with some implications for alternative therapy in poststroke limb rehabilitation.

## Methods

### Overview

The systematic review was registered a priori to PROSPERO (CRD42023488270) and reported following the PRISMA (Preferred Reporting Items for Systematic Reviews and Meta-Analyses; checklist provided in Checklist 1).

### Search Strategy

We systematically searched publications in the following databases in June 2025: PubMed, Cochrane Library, Web of Science, Embase, Proquest, Physiotherapy Evidence Database, and clinical trial registries: ClinicalTrials.gov and ChiCTR. Two researchers (PS and CN) developed and performed the search strategy together. The following keywords were used for the search: “Stroke,” “Motor Imagery,” “Action Observation,” “MI,” “AO,” etc. The search method uses the combination of Medical Subject Headings (MeSH) and free terms, and the search strategy is shown in Table 1.

**Table 1.** Search strategy.

Database	Search strategy
PubMed	1=(((Strokes[MeSH Terms]) OR (Cerebrovascular Accident*[MeSH Terms])) OR (CVA*[MeSH Terms])) OR (Apoplexy[MeSH Terms]) 2=(((((stroke*[Title/Abstract]) OR (post stroke[Title/Abstract])) OR (post‐stroke[Title/Abstract])) OR (apoplex*[Title/Abstract])) OR (Cerebrovascular Accident*[Title/Abstract])) OR (CVA*[Title/Abstract]) 3=(((((((brain[Title/Abstract]) OR (cerebr*[Title/Abstract])) OR (cerebell*[Title/Abstract])) OR (intracerebr*[Title/Abstract])) OR (intracran*[Title/Abstract])) OR (cerebral vasc*[Title/Abstract])) OR (brain vasc*[Title/Abstract])) AND ((((((((((((ischemi*[Title/Abstract]) OR (infarct*[Title/Abstract])) OR (thrombo*[Title/Abstract])) OR (emboli*[Title/Abstract])) OR (occlus*[Title/Abstract])) OR (hemorrhag*[Title/Abstract])) OR (hematoma*[Title/Abstract])) OR (bleed*[Title/Abstract])) OR (accident*[Title/Abstract])) OR (disorder*[Title/Abstract])) OR (disease*[Title/Abstract])) OR (apoplexy[Title/Abstract])) 4=((((Action observation[Title/Abstract]) OR (AO[Title/Abstract])) OR (AOT[Title/Abstract])) OR (action images[Title/Abstract])) OR (video therapy[Title/Abstract]) 5=((observ*[Title/Abstract]) OR (watch*[Title/Abstract])) AND (((((((((((action*[Title/Abstract]) OR (movement*[Title/Abstract])) OR (reach*[Title/Abstract])) OR (activit*[Title/Abstract])) OR (task*[Title/Abstract])) OR (motion*[Title/Abstract])) OR (motor[Title/Abstract])) OR (train*[Title/Abstract])) OR (perform*[Title/Abstract])) OR (gestur*[Title/Abstract])) OR (demonstrat*[Title/Abstract])) 6=(((mental[Title/Abstract]) OR (cognitive*[Title/Abstract])) OR (covert*[Title/Abstract])) AND (((((((image*[Title/Abstract]) OR (imagination[Title/Abstract])) OR (imagining[Title/Abstract])) OR (rehears*[Title/Abstract])) OR (practic*[Title/Abstract])) OR (train*[Title/Abstract])) OR (represent*[Title/Abstract])) 7=(((((motor[Title/Abstract]) OR (locomot*[Title/Abstract])) OR (visual*[Title/Abstract])) OR (motion[Title/Abstract])) OR (movement[Title/Abstract])) AND ((((image*[Title/Abstract]) OR (imagination[Title/Abstract])) OR (imagining[Title/Abstract])) OR (ideation[Title/Abstract])) 8=((((Imagery[Title/Abstract]) OR (imagination[Title/Abstract])) OR (kinesthetic imagery[Title/Abstract])) OR (Movement representation techniques[Title/Abstract])) OR (mental simulation practice[Title/Abstract]) 9=(Randomized Controlled Trial[MeSH Terms]) OR (Randomized Controlled Trials[MeSH Major Topic]) 10=((((randomized controlled trials[Title/Abstract]) OR (randomized controlled trial[Title/Abstract])) OR (RCT[Title/Abstract])) OR (RCTs[Title/Abstract])) OR (Random allocation[Title/Abstract]) 11=((control*[Title/Abstract]) OR (clinical[Title/Abstract])) AND (((trial*[Title/Abstract]) OR (stud*[Title/Abstract])) OR (experiment*[Title/Abstract])) 12=1 OR 2 OR 3 13=4 OR 5 14=6 OR 7 OR 8 15=9 OR 10 OR 11 16=12 AND 13 AND 14 AND 15
Cochrane Library	#1 (Strokes or Cerebrovascular Accident* or CVA* or Apoplexy): ti,ab,kw #2 ((brain or cerebr* or cerebell* or intracerebr* or intracran* or cerebral vasc* or brain vasc*) AND (ischemi* or infarct* or thrombo* or emboli* or occlus* or hemorrhag* or hematoma* or bleed* or accident* or disorder* or disease* or apoplexy)):ab #3 (Action observation or AO or AOT or action images or video therapy): ab,ti,kw #4 ((observ* or watch*) and (action* or movement* or reach* or activit* or task* or motion* or motor or train* or perform* or gestur* or demonstrat*)):ab #5 ((mental or cognitive* or covert*) and (image* or imagination or imagining or rehears* or practic* or train* or represent*)):ab,ti,kw #6 ((motor or locomot* or visual*or Motion or movement) and (image* or imagination or imagining or ideation)):ab #7 (Imagery or imagination or kinesthetic imagery or Movement representation techniques or mental simulation practice):ab,ti,kw #8 (randomized controlled trials or randomized controlled trial or RCT or RCTs or Random allocation):ab,ti,kw #9 ((control* or clinical) and (trial* or stud* or experiment*)):ab,ti,kw #10 #1 or #2 #11 #3 or #4 #12 #5 or #6 or #7 #13 #11 and #12 #14 #8 or #9 #15 #10 and #13 and #14
Web of Science	#1 (TS=(stroke* or post stroke or post‐stroke or apoplex* or Cerebrovascular Accident*or CVA*)) NOT (SILOID==(“PPRN”)) #2 (TS=((brain or cerebr* or cerebell* or intracerebr* or intracran* or cerebral vasc* or brain vasc*) AND (ischemi* or infarct* or thrombo* or emboli* or occlus* or hemorrhag* or hematoma* or bleed* or accident* or disorder* or disease* or apoplexy)) and Preprint Citation Index (Exclude – Database) #3 (TS=(Action observation or AO or AOT or action images or video therapy)) NOT (SILOID==(“PPRN”)) #4 (TI=((observ* or watch*) and (action* or movement* or reach* or activit* or task* or motion* or motor or train* or perform* or gestur* or demonstrat*))) NOT (SILOID==(“PPRN”)) #5 TS=((mental or cognitive* or covert*) and (image* or imagination or imagining or rehears* or practic* or train* or represent*)) and Preprint Citation Index (Exclude – Database) #6 (TI=((motor or locomot* or visual*or Motion or movement)and (image* or imagination or imagining or ideation))) NOT (SILOID==(“PPRN”)) #7 (TI=(Imagery or imagination or kinesthetic imagery or Movement representation techniques or mental simulation practice)) NOT (SILOID==(“PPRN”)) #8 TS=(randomized controlled trials or randomized controlled trial or RCT or RCTs or Random allocation) and Preprint Citation Index (Exclude – Database) #9 TS=((control* or clinical) and (trial* or stud* or experiment*)) and Preprint Citation Index (Exclude – Database) #10 #1 or #2 and Preprint Citation Index (Exclude – Database) #11 #3 or #4 and Preprint Citation Index (Exclude – Database) #12 #5 or #6 or #7 and Preprint Citation Index (Exclude – Database) #13 #8 or #9 and Preprint Citation Index (Exclude – Database) #14 #11 and #12 and Preprint Citation Index (Exclude – Database) #15 #10 and #13 and #14 and Preprint Citation Index (Exclude – Database)

### Inclusion Criteria and Exclusion Criteria

The studies were selected based on the PICOS verification method (P-participants; I-intervention; C-comparison; O-outcome; and S-study design), as shown in Table 2.

**Table 2.** Selection criteria for meta-analysis.

Category	Inclusion criteria
Participants	Adult patients with stroke (age >18 years), conscious, with motor impairment. Selection of studies was not influenced by the chronicity, severity, or type of the stroke
Intervention	A combination of action observation and motor imagery intervention with or without routine rehabilitation. Studies were included even if the AO^a^+MI^b^ intervention group was contaminated with other concurrent treatment effects, such as mirror box therapy or graded motor imagery
Comparison	Routine physical therapy or occupational therapy. AO or MI independently, with or without routine rehabilitation Placebo or no therapy
Outcome	Primary outcome: limb function. Assessment tools include but are not limited to FMA-tUE^c^, ARAT^d^, WMFT^e^, WAQ^f^, and MAL^g^ Secondary outcomes: dependence on ADL^h^, measured tools include but are not limited to the Barthel Index and FIM^i^
Study design	Clinical randomized controlled trial
Exclusion criteria	Conference articles The full text cannot be obtained Non-English literature Inability to extract outcome index data

a AO: action observation.

b MI: motor imagery.

c FMA-UE: Fugl-Meyer assessment.

d ARAT: Action Research Arm Test.

e WMFT: Wolf Motor Function Test.

f WAQ: Walking Ability Questionnaire.

g MAL: Motor Activity Log.

h ADL: activities of daily living.

i FIM: Functional Independence Measure.

### Study Selection and Data Extraction

Two reviewers (XL and XZ) carried out the study selection independently in accordance with the eligibility criteria, and in case of disagreement, a third reviewer (PS) was asked to resolve this. Initially, all potential articles were screened according to the title, abstract, and keywords. Then the full text of all selected articles was checked, and the final selection was made based on the predefined selection criteria.

Three reviewers (PS, XL, and XZ) independently extracted data from the included studies using a predefined form. Data abstraction included study characteristics, participant characteristics, sample size, intervention measures and intervention content, duration of experiments, assessment instrument, and outcomes. Pre-post differences data were extracted directly. For studies that did not report the mean and SD of the pre-post differences, we used the Follmann et al [32] method to convert the means and SDs of the baseline and final values into the mean and SD of the pre-post differences, as described in version 3.0.2 of the Cochrane Collaboration Handbook (page 213). In case of missing or unclear data, we tried to obtain additional information from the study authors.

### Risk of Bias and Certainty of the Evidence

Methodological quality assessment will be conducted independently by 2 researchers, following the Cochrane Risk of Bias tool (RoB 2.0; The Cochrane Collaboration) for assessing the risk of bias in randomized trials [33] recommended in the Cochrane Handbook [34]. If 2 reviewers (XL and XZ) cannot determine the evaluation results in the quality evaluation, a third independent reviewer (PS) makes the decision.

Two independent reviewers (XZ and XL) used the Grading of Recommendations Assessment, Development and Evaluation (GRADE) approach to assess the certainty for each meta-analysis outcome. There are 4 classification levels to assess the quality of evidence: high, moderate, low, and very low. We used the GRADEpro GDT (GRADEpro GDT; Evidence Prime, Inc) to create the “Summary of findings” tables.

### Data Analysis

Statistical analysis was performed using Review Manager software (RevMan 5.3; The Cochrane Collaboration). Measurement data with the same outcome index unit were calculated using mean difference and 95% CI. SMD and 95% CI were used for different outcome index units. The difference was statistically significant at *P*<.05. For studies reporting median, we converted these values to mean and SD using the method proposed by Hozo et al [35].

Heterogeneity was tested using the chi-square test and *I*^2^ statistic [36]. The heterogeneity was considered low when *P*>.1 and *I*^2^<50%, and the fixed-effects model estimated the combined effect size. The heterogeneity was considered significant when *P*<.1 and *I*^2^>50%, and the random effects model was adopted. When it was impossible to combine effect sizes, we performed only a descriptive analysis of the results. Subgroup analyses were conducted to explore potential sources of heterogeneity through subgroup analysis.

For interventions that could not be directly compared, a mesh meta-analysis was performed using Stata 14.1 software (StataCorp LLC). SMD and 95% CI were used to calculate the effect size of this study. The surface under the cumulative ranking curve (SUCRA) was used to predict and rank the efficacy of rehabilitation measures [37]. When there was a closed loop in the network map, the inconsistency model was used for testing. If *P*>.05, the consistency model was used for analysis. If *P*≤.05, the source of heterogeneity should be identified and eliminated.

We conducted the sensitivity analysis using the one-by-one elimination method and changing the combined model. If more than 10 studies were included, the funnel plot was used to analyze publication bias.

## Results

### Study Selection and Characteristics

**Figure 1.**
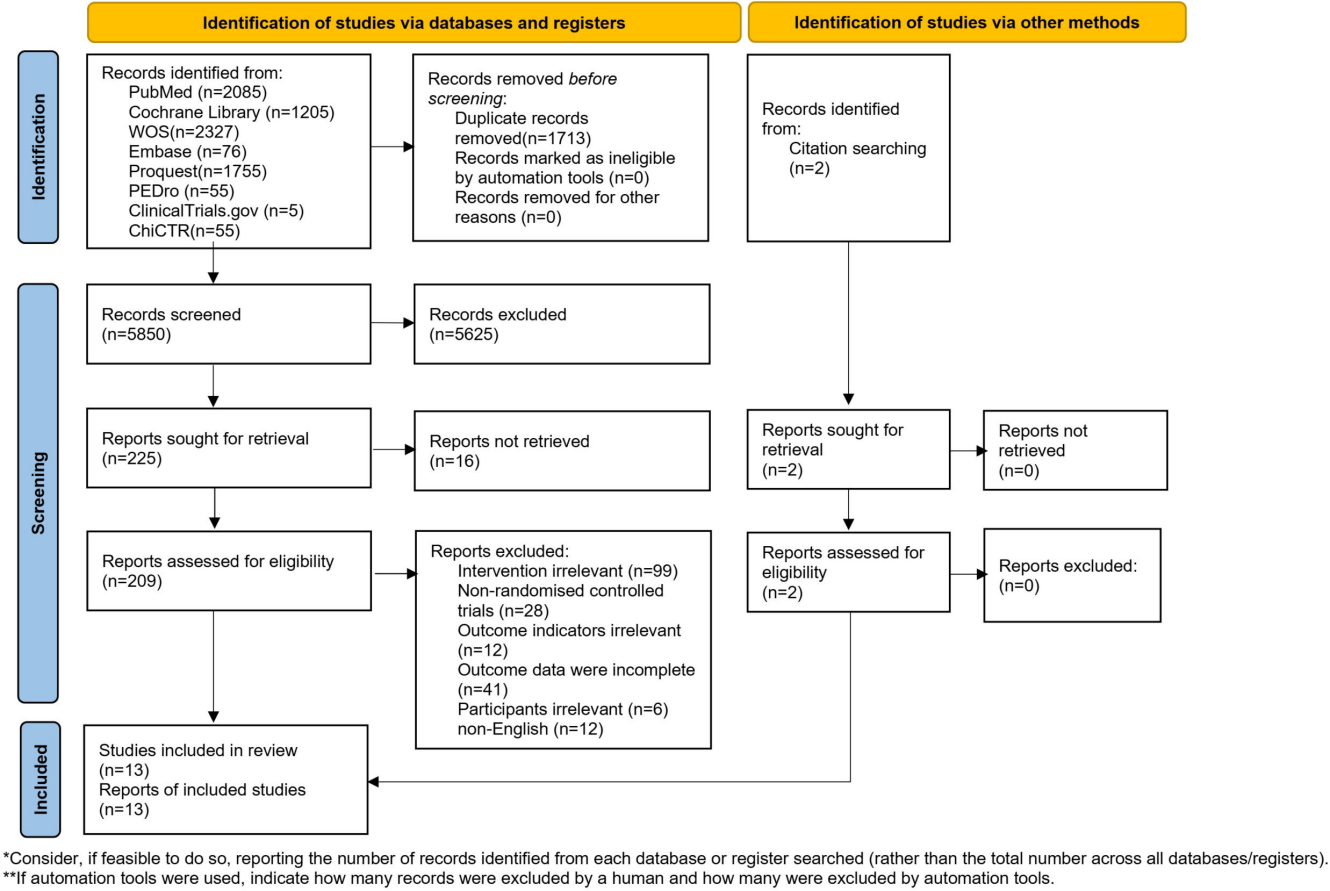
PRISMA (Preferred Reporting Items for Systematic Reviews and Meta-Analyses) flowchart of literature screening (source: adapted from Page and Levine [47], which is published under Creative Commons Attribution 4.0 International License [51]).

A total of 7563 results were retrieved from 8 databases, and 5850 results remained after removing duplicates. After the preliminary screening, 219 articles remained. After reading the full text and rescreening, 13 articles [38-50] were finally included, and the flowchart is shown in Figure 1.

The 13 included studies [38-50] involved a total of 399 participants, and most of them were small sample trials. The participants were mainly patients with stroke with upper limb paralysis. A total of 12 studies compared AO+MI with their independent use or conventional rehabilitation [38-49]. In addition, one [50] compared synchronous and asynchronous combination models. Most of these studies had an intervention duration of about 4 weeks. Most of the primary outcome indicators were upper extremity function, while only 2 studies [39,45] assessed the lower extremity function and 1 study [49] assessed the overall motor function. The features of literature inclusion are shown in Table 3. In this table, age and stroke course were expressed as mean (SD) or median (IQR) or median (maximum, minimum).

**Table 3.** Characteristics of included studies.

Study	Country	Participants	Sample size (n)	Age (years)	Intervention	Duration of experiment	Outcomes measure	Summary of results
Green et al 2023 [41]	United States	≤1 month after stroke onset paralysis of one upper limb	EG: G1: 5; G2: 4 CG: G3: 4; G4: 5	EG: G1: 71.4±7.1; G2: 58.5±8.8 CG: G3: 59.0±11.5; G4: 60.8±11.2	EG: G1: MI^a^+audio and repetitive task practice; G2: asynchronous AO (video)+MI^b^ and repetitive task practice; CG: G3: repetitive task practice; G4: conventional rehabilitation. The intervention tasks for group 1, group 2, and group 3 included (a) wiping a table, (b) picking up a cup, (c) brushing hair, and (d) turning the page of a book. The intervention tasks for group 4 included range of motion, weight-bearing, massage, modalities, and task-oriented training.	During hospitalization 20 rounds of mental practice and 10 body exercises per session, 3 times a week 20 rounds of mental practice only, 2 times a week	WMFT^c^ FMA-UE^d^	A significant change in FMA-UE scores and WMFT time scores in the G1 and G4, but no significant change in the G2 and G3 groups. Among all groups, no statistically significant change was found between pretest and posttest scores for the WMFT functional ability score.
Rungsirisilp et al 2023 [48]	Thailand	≥6 months after stroke onset Upper limb paralysis	EG: 9 CG: 8	EG: 61.11±7.16 CG: 61.63±7.83	EG: Synchronous AO+MI-based BCI^e^ and physical therapy. CG:MI-based BCI and physical therapy. The AO+MI or MI tasks included wrist and hand extensions.	AO+MI/MI: 40 trials per session, 3 times a week, for 4 weeks Physical therapy: 1 to 2 days a week	FMA-UE	Both AO+MI and MI-based BCI training improved upper limb function in patients with chronic stroke. However, the EG showed significantly greater motor gain than the CG.
Sui et al 2023 [49]	China	2 weeks to 3 months after stroke onset With motor dysfunction	EG: 50 CG: 50	EG: 59.50±4.80 CG: 58.90±4.78	EG: asynchronous AO+MI and conventional rehabilitation. CG: conventional rehabilitation. The AO+MI tasks included (a) steady trunk movement with a Bobath ball and (b) balance movements while sitting, standing, and reaching out to move a water cup. The conventional rehabilitation training included good limb positioning, neuromuscular promotion techniques, such as the proprioceptive neuromuscular facilitation (PNF) technique, Rood approach, motor relearning, occupational therapy, daily living ability training, and traditional therapy.	AO+MI: 30 minutes per session, 5 times a week, for 4 weeks Conventional rehabilitation: 5 hours per day, 5 days a week for 4 weeks	FMA	After treatment, FMA in the 2 groups was significantly higher than those before treatment, with the EG showing higher scores than the CG.
Choi et al 2022 [40]	Korea	2‐8 months after stroke onset Upper limb paralysis	EG: 22 CG: 23	EG: 62.68±8.54 CG: 63.43±9.57	EG: synchronous AO+MI and physical therapy. CG:AO and physical therapy. The AO+MI or MI tasks included 10 ADLs^f^ (eg, using chopsticks, pen, and hand washing). Participants selected and completed 5 meaningful activities.	AO+MI/AO: 25 minutes per session, 5 times a week, for 8 weeks Physical therapy: 30 minutes each day	FMA-UE WMFT MAL^g^	The EG showed significant improvements in FMA-UE, WMFT, and MAL scores after the intervention compared to preintervention and the CG.
Page et al 2021 [47]	United States	≥3 months after stroke onset Hand dyskinesia	EG: 9 CG: 9	EG: 57.4±10 CG: 57.8±9.8	EG: asynchronous AO+MI and repetitive task practice. CG: repetitive task practice. The repetitive task practice and AO+MI tasks included 5 upper extremity activities.	3 times a week for 10 weeks EG: 30 minutes of AO+MI and 15 minutes of occupational therapy CG: 45 minutes of occupational therapy	FMA-UE ARAT^h^ SIS-H^i^	The EG exhibited significantly larger increases on all 3 outcome measures compared with the CG and surpassed minimal clinically important difference standards for all 3 UE outcome measures.
Nam et al 2019 [46]	Korea	1‐6 months after stroke onset Wrist extensor below grade 2	EG: 10 CG: 10	EG: 61.8±14.0 CG: 59.6±15.0	EG: synchronous AO+MI and conventional rehabilitation. CG: conventional rehabilitation. The AO+MI tasks included grasping, carrying a cube, pegboard, pinching, sliding an object across a table, and holding a cup. The conventional rehabilitation therapy included proprioceptive exercises, muscle strengthening, gait training, and paretic hand and wrist mobilization, among others.	5 times a week for 4 weeks EG: 20 minutes of AO+MI and 30 minutes of conventional rehabilitation per session CG: 50 minutes of conventional rehabilitation per session	FMA-UE MFT^j^ FIM^k^	Significant differences were found from baseline to postintervention assessments within both groups on FMA-UE and FIM scores, whereas there were no statistically significant differences in mean FMA-UE, MFT, and FIM scores between groups.
Kim et al 2018 [43]	Korea	≥3 months after stroke onset Brunnstrom stage ≥3	EG: 7 CG: 7	EG: 52 (49–74) CG: 66 (49–72)	EG: asynchronous AO+MI. Modified constraint-induced motion. CG: listen to music. Modified constraint-induced motion. The AO+MI task was to visualize the use of a spoon to eat soup with the affected hand. Modified constraint-induced motion included 5 to 6 tasks, repetitive ADL tasks (eg, brushing teeth, drinking from a cup, making a phone call).	AO+MI/Listen to music: 10 minutes per session, 5 times a week for 2 weeks Modified restraint-induced movement for more than 6 hours every day	JTHFT^l^ MAL	Both groups showed significant improvement in the MAL scores. The EG also showed significant improvement in the JTHFT scores. The improvements in the JTHFT and MAL scores were significantly greater in the EG compared to the CG.
Sun et al 2016 [50]	China	≤2 months after stroke onset Hemiplegia of the right upper limb	EG: 5 CG: 5	59.8±4.94	EG: synchronous AO+MI and conventional rehabilitation. CG: asynchronous AO+MI and conventional rehabilitation. The AO+MI task was having the participant use his (or her) right arm to insert a peg into the hole on a wooden board and then to remove it from the board.	15 rounds AO+MI each time, 6 times a day, 7 days a week for 4 weeks	FMA-UE PST^m^	The FMA and PST scores achieved with the synchronous AO+MI group were also significantly higher than those achieved with the asynchronous group.
Assis et al 2016 [38]	Brazil	≥12 months after stroke onset Grade 2 spasm at most	EG: 4 CG: 4	EG: 50.5 CG: 59.5	EG: asynchronous AO+MI based on augmented reality and conventional rehabilitation. CG: conventional rehabilitation. The AO+MI tasks included shoulder abduction, shoulder flexion, and shoulder horizontal flexion.	60 minutes per session, once a week, for 4 weeks	FMA-UE	At a significance level of 5%, Fugl-Meyer scores suggested a trend for greater upper-limb motor improvement in the EG than in the CG.
Lee et al 2013 [44]	Korea	≥6 months after stroke onset Brunnstrom stage 5	EG: 8 CG: 7	EG: 63±3.7 CG: 60±5.9	EG: synchronous AO+MI. CG: sham control. The AO+MI task was the action of stretching out the right hand to pick up a cup, bringing the cup to the mouth in order to touch the mouth, and then returning the cup to its initial position.	10 minutes per session, 5 times a week, for 3 weeks	Number of drinking motions in each period	The drinking behavior functions for the EG showed a significant difference before and after the intervention, and the postintervention performance of the EG was superior to that of the CG.
Cho et al 2013 [39]	Korea	≥6 months after stroke onset Ability to walk >10 m independently	EG: 15 CG: 13	EG: 53.93±12.60 CG: 53.85±12.44	EG: synchronous AO+MI and gait training. CG: gait training. The AO+MI task was the normal gait movement.	3 times a week, for 6 weeks EG:15 minutes of AO+MI and 30 minutes of gait training CG: 30 minutes of gait training	FMA-LE^n^ TUG^o^ FRT^p^	All measurements improved significantly compared with baseline values in the EG. In the CG, there were significant improvements in all parameters except the Fugl-Meyer assessment.
Lee et al 2011 [45]	Korea	≥6 months after stroke onset Ability to walk >10 m independently	EG: 13 CG: 11	EG: 60.7±7.35 CG: 61.9±11.26	EG: asynchronous AO+MI and gait training. CG: gait training. The AO+MI task was the normal gait movement.	3 times a week, for 6 weeks EG: 30 minutes of AO+MI and 30 minutes of gait training CG: 30 minutes of gait training	Gait speed	The gait speed among the temporal parameters had significantly increased in the EG, compared to preintervention.
Ietswaart et al 2011 [42]	United Kingdom	1‐6 months after stroke onset Persistent arm movement weakness	EG: 39 CG: G1:31; G2: 32	EG: 69.3±10.8 CG: G1: 68.6±16.3; G2: 64.4±15.9	EG: asynchronous AO+MI. CG: G2: attention-placebo control intervention. G2: sham control. The AO+MI tasks included a variety of elementary movements (eg, opening and closing of the hand), goal-directed movements (eg, reaching), and activities of daily living (eg, doing buttons on a shirt). Attention-placebo refers to cognitive training that does not involve motor imagery, including sustained attention, visualization, memory demands, visual illusions, and inhibition.	EG: 30 minutes of AO+MI, 10 minutes of active MI, and 5 minutes of a certain implicit form of MI per session, 3 times a week, for 4 weeks G2: 25 minutes of active visual and sensory imagery, 10 minutes of controlling for cognitive inhibition, 5 minutes of watching optical illusions of motion, and 5 minutes of a visual imagery activity per session, 3 times a week, for 4 weeks	ARAT Grip strength Barthel Index	Recovery between baseline and outcome assessment was evident on all outcome variables. However, no differences between the 3 groups were found on the ARAT scores, grip strength, and Barthel Index.

a MI: motor imagery.

b AO+MI: action observation combined with motor imagery.

c WMFT: Wolf Motor Function Test.

d FMA-UE: Fugl-Meyer assessment of upper extremity.

e BCI: brain–computer interface.

f ADL: activities of daily living.

g MAL: Motor Activity Log.

h ARAT: Action Research Arm Test.

i SIS-H: hand subscale of the Stroke Impact Scale.

j MFT: manual function test.

k FIM: Functional Independence Measure.

l JTHFT: Jebsen-Taylor hand function test.

m PST: pinch strength test.

n FMA-LE: Fugl-Meyer assessment lower extremity.

o TUG: Timed Up and Go Test.

p FRT: Functional Reaching Test.

### Risk of Bias and Certainty of the Evidence

**Figure 2.**
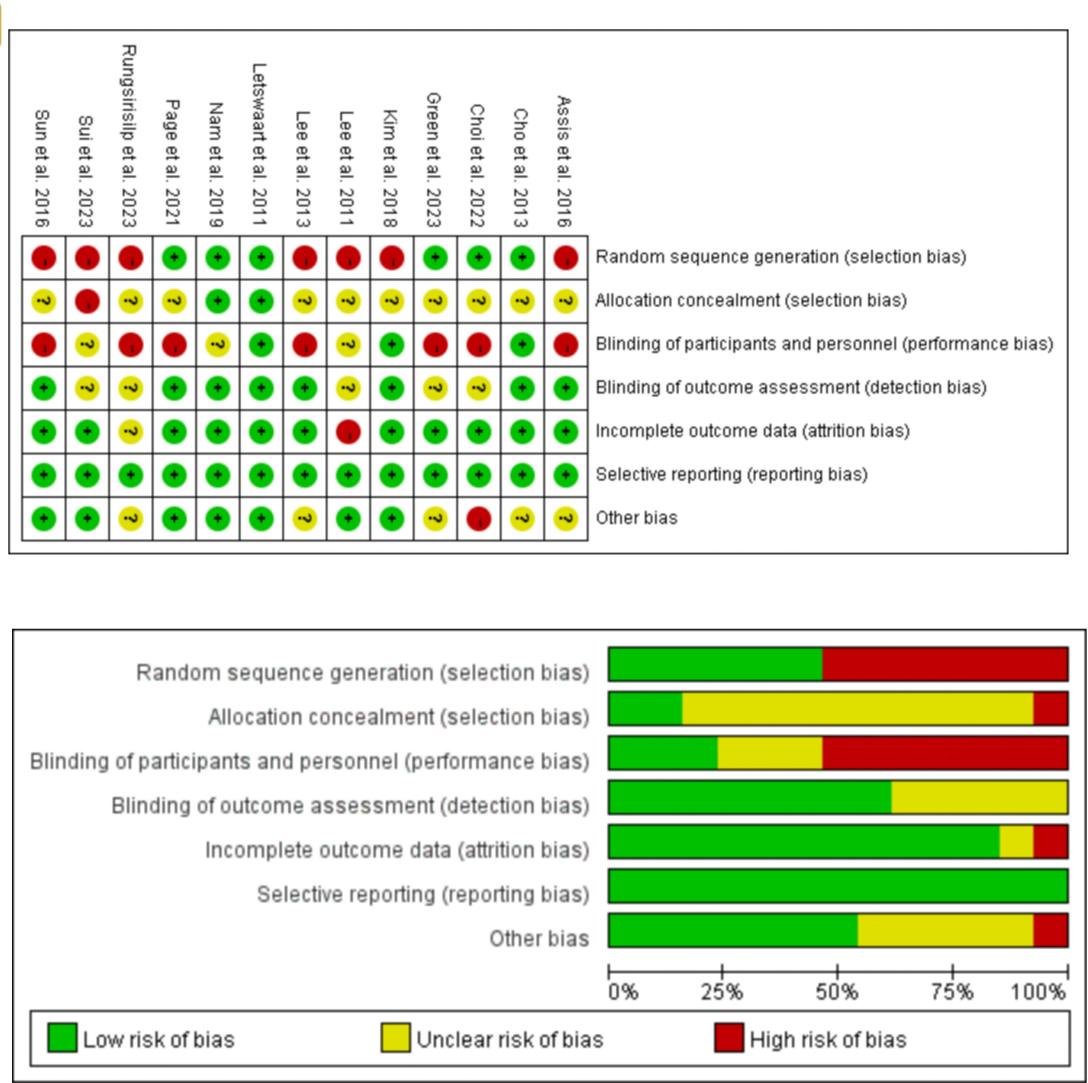
Risk of bias graph [38-50].

The RoB 2.0 tool was used to evaluate literature quality. All 13 randomized controlled studies were included, of which 6 [39-42,46,47] had a low risk of selection bias, 2 [42,46] used assignment concealment, 3 [39,42,43] blinded participants and implementors, 8 [38,39,42-44,46,47,50] blinded outcome evaluators, only one [48] outcome measure was incomplete, all studies reported full results, and 7 [42,43,45-47,49,50] had a low risk of other bias. The final evaluation results are shown in Figure 2.

We assessed the certainty of evidence for the 7 outcomes in the meta-analysis. The overall certainty of evidence was rated as low to very low. The GRADE results are shown in Table 4.

**Table 4.** Summary of findings.

Certainly assessment							No. of patients		Effect	
No. of studies	Study design	Risk of bias	Inconsistency	Indirect evidence	Imprecision	Others	Experimental	Control	Absolute (95% CI)	Certainty
Overall motor function (AO+MI^a^ vs routine)										
1	RCTs^b^	Serious^c^	Not serious	Not serious	Serious^d^	None	50	50	SMD^e^ 3.22 higher (1.55 to 4.89 higher)	⊕⊕◯◯ Low
Upper extremity function (AO+MI vs routine)										
7	RCTs	Not serious	Serious^f^	Serious^g^	Serious^d^	None	81	76	SMD 1.02 higher (0.28 to 1.75 higher)	⊕◯◯◯ Very low
Self-care ability (AO+MI vs routine)										
2	RCTs	Not serious	Not serious	Not serious	Very Serious^h^	None	49	42	SMD 0.06 higher (0.35 lower to 0.47 higher)	⊕⊕◯◯ Low
Lower extremity function (AO+MI vs routine)										
2	RCTs	Serious^c^	Not serious	Not serious	Serious^d^	None	28	24	SMD 6.31 higher (4.75 to 7.87 higher)	⊕⊕◯◯ Low
Upper extremity function (AO+MI vs MI)										
2	RCTs	Serious^c^	Not serious	Not serious	Serious^d^	None	13	13	SMD 0.97 higher (0.13 to 1.80 higher)	⊕⊕◯◯ Low
Upper extremity function (AO+MI vs AO)										
1	RCTs	Serious^c^	Not serious	Not serious	Serious^d^	None	22	23	SMD 1.92 higher (0.66 to 3.18 higher)	⊕⊕◯◯ Low
Upper extremity function (asynchronous vs synchronous)										
9	RCTs	Serious^c^	Not serious	Serious^i^	Very Serious^h^	None	93	22	SMD 1.00 higher (1.33 lower to 3.33 higher)	⊕◯◯◯ Very low

a AO+MI: AO combined with MI.

b RCT: randomized controlled trial.

c 50%＜*I*2＜75%.

d One study used surrogate endpoint measures to replace the patient’s important outcome of interest.

e SMD: standard mean difference.

f The sample size was less than 100.

g The 95% CI crosses the equivalence line and the sample size is less than 100.

h Most of the information (two-thirds) came from moderate bias.

i Network meta-analysis.

### Outcomes

#### Comparison of AO+MI and Routine Rehabilitation

**Figure 3.**
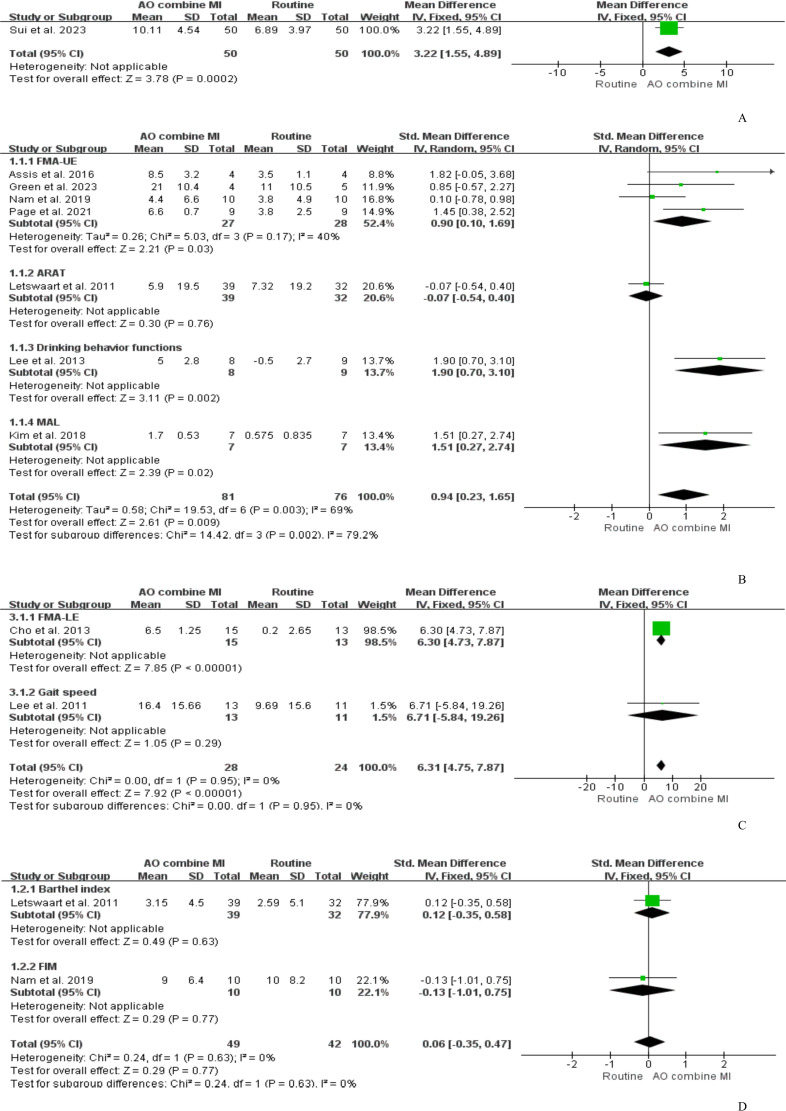
The action observation combined with motor imagery therapy versus conventional physical therapy for (A) overall motor function, (B) upper extremity motor function, (C) lower extremity motor function, and (D) activities of daily living [38,39,41-47,49].

A total of 10 studies compared the effects of AO+MI and routine rehabilitation on patients’ motor function [38,39,41,42,44-47,49,50]. One study [49] evaluated the impact of AO+MI on overall motor function, demonstrating that AO+MI enhanced Fugl-Meyer assessment (FMA) scores versus routine rehabilitation (SMD=3.22, 95% CI 1.55‐4.89; *P*<.001), as shown in Figure 3A.

A total of 7 studies [38,40,41,46-48,50] assessed the effect of AO+MI on upper extremity motor function and were included in the meta-analysis. Among these 7 studies, 4 studies [38,41,46,47] used Fugl-Meyer assessment of upper extremity (FMA-UE) as the main evaluation index to evaluate the upper extremity motor function of patients, one study [42] used Action Research Arm Test (ARAT), one study [43] used Motor Activity Log (MAL), and one study [44] used the data of 1-minute drinking movements of patients. A random effects model was used as the studies had high heterogeneity (*P*=.002, *I*^2^=71%). The results showed a significant difference between the 2 groups (SMD=1.02, 95% CI 0.28‐1.75; *P*=.007), as shown in Figure 3B.

A total of 2 studies [39,45] evaluated the effects on lower extremity motor function, with one study [39] using the Fugl-Meyer assessment lower extremity (FMA-LE) as the evaluation index and one study [45] using gait speed. Analysis using a fixed-effects model (*P*=0.95, *I*^2^=0%) and meta-analysis results showed a significant difference between the 2 groups (SMD=6.31, 95% CI 4.75‐7.87; *P*<.001), as shown in Figure 3C.

A total of 2 studies [42,46] evaluated the effects on activities of daily living (ADL), with one study [42] using the Barthel index as the evaluation index and one study [46] using the Functional Independence Measure (FIM). Analysis using a fixed-effects model (*P*=0.63, *I*^2^=0%) and meta-analysis results showed no statistically significant difference in ADL between the 2 groups (SMD=0.06, 95% Cl −0.35 to 0.47; *P*=.77), as shown in Figure 3D.

#### Comparison of AO+MI and Their Independent Use

**Figure 4.**
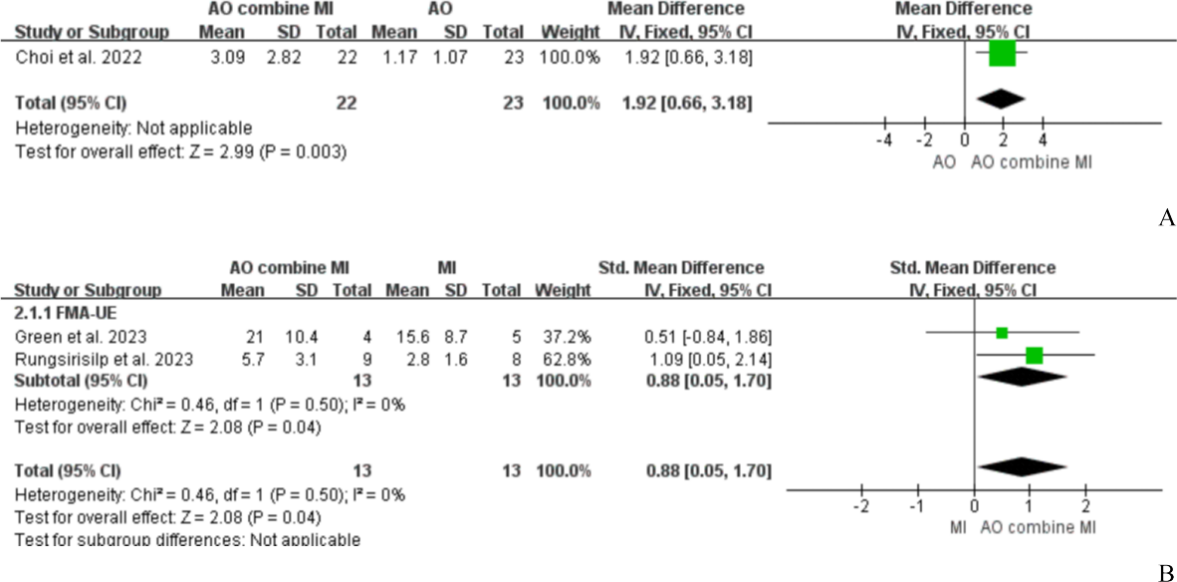
A, Action observation combined with motor imagery versus action observation independently for upper extremity function and B, AO+MI versus motor imagery independently for upper extremity function [40,41,48]. AO: action observation, AO+MI: Action observation combined with motor imagery, FMA-UE: Fugl-Meyer assessment of upper extremity, MI: motor imagery.

A total of 3 studies [40,41,48] compared the effects of AO+MI and their independent use on patients’ motor function. One study [40] compared the effects of AO+MI and AO on patients’ upper extremity function. The results demonstrated a statistically significant improvement in FMA-UE scores with AO+MI relative to AO alone (SMD=1.92, 95% CI 0.66‐3.81; *P*=.003), as shown in Figure 4A. Two studies [41,48] compared the effects of AO+MI and MI independently on patients’ upper extremity function; both used FMA-UE as an evaluation index. A meta-analysis was performed using a fixed-effects model (*P*=.69, *I*^2^=0%), and results showed that AO+MI could promote the recovery of upper extremity function in patients compared with MI independently (SMD=0.97, 95% Cl 0.13‐1.80; *P*=.02), as shown in Figure 4B.

#### Comparison of Synchronous Mode and Asynchronous Mode

**Figure 5.**
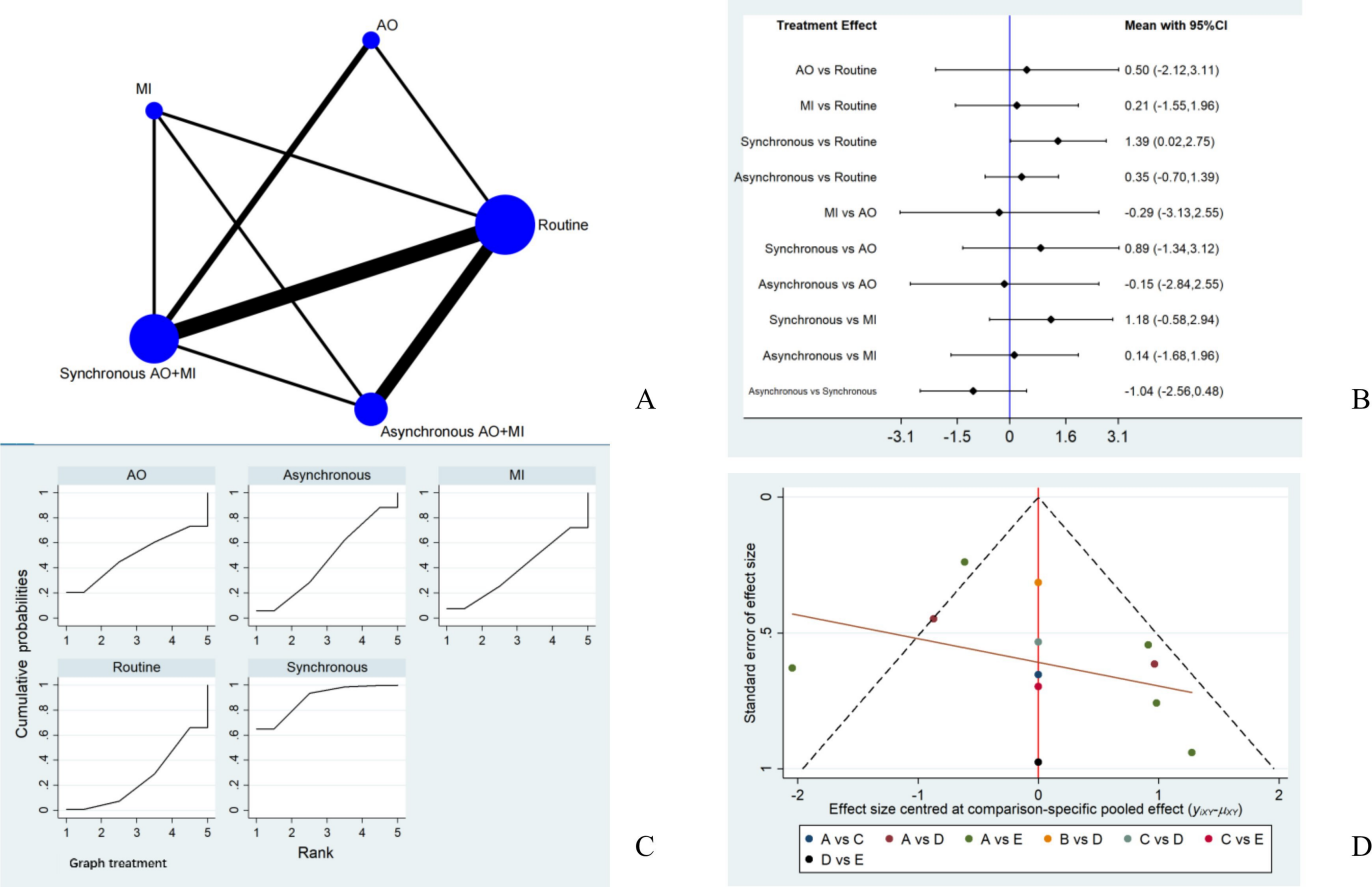
(A) The network diagram. (B) Forest plot of the network meta-analysis for upper extremity function. (C) Surface under the cumulative ranking curve analyzes the sort diagram. (D) The comparison-correction funnel plot. AO: action observation; AO+MI: action observation combined with motor imagery; MI: motor imagery.

Of the studies included in this review, 7 studies [38,41-43,45,47,49] used the asynchronous AO+MI mode, 5 studies [39,40,44,46,48] used synchronous mode, and one study [50] directly compared the therapeutic effects of synchronous and asynchronous combined mode. A total of 10 studies [38,40-44,46-48,50] were included in the network meta-analysis to compare the effects of the 2 combination interventions on upper extremity motor function across 5 rehabilitation measures: routine rehabilitation, AO, MI, synchronous AO+MI and asynchronous AO+MI; the network diagram is shown in Figure 5A. FMA-UE, ARAT, MAL, and 1-minute drinking movements of patients were used as indices to evaluate extremity function rehabilitation.

The global inconsistency test revealed no inconsistency between the studies (*χ*^2^df=1.53, *P*=.67). The results of the node-splitting analysis demonstrated local inconsistency between synchronous mode and asynchronous mode (*P*=.02). The forest map results showed no statistically significant difference between synchronous mode and asynchronous mode effects on upper extremity function rehabilitation of patients after stroke (SMD=−1.04, 95% Cl −2.56 to 0.48), as shown in Figure 5B. The results of the SUCRA analysis showed that the interventions most likely to promote the rehabilitation of patients’ upper extremity function were asynchronous (89.3), synchronous (46.4), AO (49.9), MI (38.6), and routine rehabilitation (25.8), as shown in Figure 5C.

#### Publication Bias

The analysis of the effect index for limb motor function was conducted, generating a funnel plot. The results showed good symmetry, indicating that publication bias was not noticeable. The comparison-correction funnel plot is shown in Figure 5D.

## Discussion

### Principal Findings

This study review aimed to synthesize the evidence on the effects of AO+MI on limb functional rehabilitation in patients after stroke through a systematic review and meta-analysis. We included 13 trials with 399 participants in this review.

The meta-analysis showed that compared with routine rehabilitation therapy, AO+MI could improve limb motor function in patients with stroke. And compared with their independent use, AO+MI could improve upper extremity motor function in patients with stroke. These findings are consistent with the results of a meta-analysis by Chye et al [21] that included all populations (encompassing patients with Parkinson disease, older individuals, children, and healthy adults et al). Relevant and experience-dependent practice encourages the brain to create and reorganize functionally appropriate neural connections, which is pivotal for neurorehabilitation following stroke [52]. A related functional magnetic resonance imaging study [53] has revealed that when individuals engage in AO+MI, blood oxygen level-dependent (BOLD) signals increase and become more widespread in brain regions involved in motor execution. Electroencephalography investigation demonstrates that during AO+MI, individuals exhibit significantly decreased power spectral densities in the alpha and beta bands, indicating heightened activity in the primary sensorimotor cortices [54]. For patients with stroke who have difficulty performing actual movements, the increased neural activity during AO+MI may support repeated Hebbian modulation of intracortical and subcortical excitatory mechanisms through synaptic plasticity, producing effects similar to those of physical practice [21]. This may be the reason why AO+MI improves motor function in patients with stroke. However, our meta-analysis showed that AO+MI did not improve ADL in patients after stroke compared with routine rehabilitation. A potential explanation is that poststroke ADL improvement requires a longer intervention duration, while the 2 studies [42,46] included in this meta-analysis featured relatively short intervention periods (2‐4 weeks).

Although the only direct evidence suggests that synchronous may enhance motor function in patients with stroke compared to asynchronous [50], the network meta-analysis results in this review indicate no significant difference between synchronous and asynchronous AO+MI in improving upper extremity function in patients with stroke. The potential reason for the inconsistency between direct evidence and meta-analysis results may stem from differences among participants. As Eaves and colleagues point out [5], synchronous AO+MI may impose greater cognitive load compared to asynchronous AO+MI. This could result in the synchronous mode being overly demanding for certain patient populations or rehabilitation stages. In such cases, asynchronous intervention might be more advantageous. Consequently, variations in key characteristics among participants across included studies—such as severity of neurological deficits and baseline cognitive levels—may modulate the boundaries of effectiveness between synchronous and asynchronous intervention modalities. Therefore, future studies with more direct evidence are needed to compare the effects of these 2 modalities on poststroke recovery. In addition, asynchronous AO+MI does not rule out the possibility of participants spontaneously performing MI during the AO component of AO then MI. This may result in some studies using asynchronous mode inadvertently including elements of synchronous mode, which might also explain why no significant difference in effectiveness has been observed between the 2 combined approaches.

This review evaluated the included studies strictly according to the relevant measures in the Cochrane 5.1.0 manual for randomized controlled trial quality assessment. Among the 13 studies [38-50] included, only one study [42] had a low risk of bias in all 7 aspects of bias analysis, while the risk of bias in the other studies mainly focused on selection bias and implementation bias. Although most studies claim randomization, only half clearly state the correct way to generate random sequences. In addition, most studies did not report how the randomization scheme was assigned to hide, which led to high-risk outcomes in both evaluations. Most of the studies included in this review were not blinded, which may be due to the changes in rehabilitation measures and the limitations of human resources, material resources, and venues, making it challenging to perform double blindness. The overall quality of the evidence presented in this review, as assessed by the GRADE approach, was low or very low for all comparisons. The main limitations were imprecision due to very small sample sizes, risk of bias, and inconsistency.

In addition to the low quality of the included articles, this review has several limitations. First, the studies we included may exhibit a certain degree of heterogeneity in terms of interventions. In some studies, the effects of AO +MI might be influenced by other concurrent therapeutic approaches, such as mirror therapy or graded motor imagery. For instance, in the study by Ietswaart et al [42], in addition to the 30-minute AO+MI intervention, each session also included an additional 10 minutes of active motor imagery and 5 minutes of a certain implicit form of motor imagery activity. This multicomponent intervention approach may mean that the observed therapeutic effects cannot be entirely attributed to pure AO+MI, potentially affecting the results of this study in the overall analysis. Second, there are a few included articles, and most are small sample studies, thus limiting the reliability of the research conclusions. Third, we only searched and reviewed articles in English; publications in other languages may have been missed. Fourth, the absence of standardized intervention protocols and variation in outcome assessment tools across the included studies complicates direct comparisons and meaningful synthesis of the findings. In addition, significant heterogeneity was observed in the meta-analysis results. Consequently, the results should be interpreted cautiously.

Dorsch et al 2024 [55] meta-analysis explored the effect of adding nonstimulation-based priming prior to task-specific practice on activity and motor impairment outcomes compared with task-specific practice alone in stroke rehabilitation, searching for manuscripts up to March 2019. This review included 2 trials investigating the impact of AO+MI on activity outcomes in patients after stroke, and the results did not show a significant improvement effect of AO+MI. The reason for the inconsistency with our findings might be that Dorsch et al [55] compared the postintervention scores between the conventional group and the AO+MI group, whereas our study compared the pre-post differences of the 2 groups.

Lin et al [56] systematic review included 9 studies investigating the effects of AO+MI on upper limb function in patients with stroke, and the results demonstrated that AO+MI significantly improves upper limb function in participants. Our study differs from Lin et al [56] review in several key aspects. First, our study adopted broader inclusion criteria, examining not only upper extremity function but also lower extremity function, overall motor function, and activities of daily living, thereby addressing a wider range of clinical outcomes. As a result, the set of included studies differs somewhat from that in Lin et al [56] review. Second, this study conducted separate meta-analyses according to the type of control group (conventional intervention, MI alone, or AO alone), thereby minimizing heterogeneity introduced by differences in control conditions, enhancing the precision of the findings, and offering more specific guidance for clinical practice. In addition, this study incorporated a network meta-analysis to compare the effectiveness of 2 AO+MI integration modes—synchronous versus asynchronous—on upper extremity function after stroke. Therefore, although there are overlapping findings, this study and Lin et al [56] review can complement each other and reinforce key conclusions, serving the valuable purpose of replication and strengthening the evidence base.

### Conclusions

This systematic review and meta-analysis examined the effects of AO+MI on motor function in patients with stroke. Our results found that AO+MI can improve the motor function compared to routine rehabilitation, AO, or MI. Furthermore, no conclusive evidence supports AO+MI’s efficacy for improving ADL, nor demonstrates differential effects between synchronous and asynchronous application modes on poststroke limb function. The quality of GRADE‐based evidence in this review varied from low to very low, due to the high risk of bias and small sample sizes. Consequently, large-scale randomized controlled trials with rigorous methodology are imperative to establish definitive clinical recommendations.

## Supplementary material

10.2196/75705 Checklist 1 PRISMA checklist.

## Abbreviations

ADL: activities of daily living
AO: action observation
AO+MI: action observation combined with motor imagery
ARAT: Action Research Arm Test
BOLD: blood oxygen level dependent
FIM: Functional Independence Measure
FMA: Fugl-Meyer assessment
FMA-LE: Fugl-Meyer assessment lower extremity
FMA-UE: Fugl-Meyer assessment of upper extremity
GRADE: Grading of Recommendations Assessment, Development and Evaluation
MAL: Motor Activity Log
MeSH: Medical Subject Headings
MI: motor imagery
PRISMA: Preferred Reporting Items for Systematic Reviews and Meta-Analyses
RoB: Cochrane Risk of Bias tool
SMD: standardized mean difference
SUCRA: surface under the cumulative ranking curve
